# Levels of Contamination by Pesticide Residues, Polycyclic Aromatic Hydrocarbons (PAHs), and 5-Hydroxymethylfurfural (HMF) in Honeys Retailed in Europe

**DOI:** 10.1007/s00244-022-00970-3

**Published:** 2023-01-02

**Authors:** Magdalena Surma, Anna Sadowska-Rociek, Anna Draszanowska

**Affiliations:** 1 Department of Plant Products Technology and Nutrition Hygiene, Faculty of Food Technology, University of Agriculture, Ul. Balicka 122, 30-149, Kraków, Poland; 2 Department of Human Nutrition, The Faculty of Food Science, University of Warmia and Mazury in Olsztyn, Ul. Słoneczna 45 F, 10-710, Olsztyn, Poland

## Abstract

Honey is consumed worldwide because of its nutritional, therapeutic and medicinal properties. Generally, honey should reach the consumer in a pure form, but it is often contaminated in various ways. Thus, this study was designed to check for the presence of pesticide residues, polycyclic aromatic hydrocarbon (PAH) levels, and the content of 5-hydroxymethylfurfural (HMF) in selected European honeys (26 samples) obtained from shops. The most frequently detected organochlorine pesticide (OCPs) was 4,4’-DDD, which was found in fourteen honey samples. Slovakian rapeseed honey was most polluted due to the presence of eight OCPs. The presence of organophosphorus pesticides (OPs) was detected in all the analysed samples, and at least one OP in each tested sample exceeded the acceptable limit. PAHs were detected in most of the analysed samples. As for PAH4s, benzo[a]pyrene was found in forest honey from Slovakia and in Polish lime tree honey, whereas wild flower honey from the UK contained the highest level of PAH4. The investigated honeys from Spain and France met the requirements for HMF content, while honeys of Slovak, Italian and Polish origin in most cases exceeded the established levels (40 mg/kg).

**Supplementary Information:**

The online version contains supplementary material available at 10.1007/s00244-022-00970-3.

Bees make honey from nectar, pollen or honeydew, which they collect from different plants (Chiesa et al. 2016; Shapla et al. 2018). It has multifaceted nutritional and medicinal values (Souza Tette et al. 2016); however, its quality is affected by the floral origin of the nectar (Ben Mukiibi et al. 2021). Honey contains about 300 substances, e.g., carbohydrates, proteins, lipids, vitamins, enzymes, phenolic acids, volatile chemicals, flavonoids, organic acids, amino acids, minerals, etc. Therefore, it has healing properties (antibacterial, antifungal, antioxidant) and is the source of many trace minerals that are necessary for human health. With a global production exceeding 1 million metric tons annually, honey is a highly valued food that is widely used for both nutritional and medicinal purposes (Hrynko et al. 2018).

However, honeybees may bring to the hive numerous contaminants deposited on the plants they visit, including pesticides (plant protection products, PPPs) and other pollutants. Thus, honeybees are good biological indicators due to two factors: analyte content of bees that died as a result of pesticide poisoning, and the residues present in their bodies or in beehive products that may be detected by laboratory analyses (Bargańska et al. 2011). Furthermore, checking for pesticides in honey can provide information about the use of pesticides in and near crop fields (Rissato et al. 2007). PPPs are widely used in agriculture to protect crops. Due to their different chemical structures, pesticides belong to different classes and chemical groups, e.g., organochlorine, organophosphorus, carbamates, etc. (Chiesa et al. 2016). Although PPPs protect agricultural crops, their overuse and incorrect use can pose a threat to human health and the environment (Blasco et al. 2011; Zanella et al. 2008). Even if small amounts of pesticide residues remain in the food supply, they constitute a potential risk for human health because of their subacute and chronic toxicity (Mukherjee 2009; Rissato et al. 2006). Since some pesticides are carcinogenic and others can cause dysfunctions in the nervous and reproductive systems, they can be extremely harmful to human health, even at low concentrations (Neufeld 2000; Sharma et al. 2010). What is more, pesticide contamination of beebread, beeswax, and honey can also affect colonies’ vitality when contaminated matrices are present during larvae development, which leads to serious ecotoxicological issues (El Agrebi et al. 2020; Orantes-Bermejo et al. 2010). The large-scale application of pesticides in agriculture and horticulture can lead to mass mortality among bees, and the chemicals find their way into bee products (Bargańska and Namieśnik 2010).

Polycyclic aromatic hydrocarbons (PAHs), one of the so-called persistent organic pollutants, are another honey contaminant due to their common occurrence in the environment. These compounds, which are formed due to incomplete combustion of organic matter, are emitted into the air and can be transported over long distances. They are characterized by high toxicity, very high durability, low water solubility, and the ability to accumulate in the soil environment (Oleszczuk 2006, 2007). Low molecular weight (LMW) PAHs have three or less aromatic rings, while high molecular weight (HMW) PAHs have four or more rings. HMW pose the greatest threat to the environment, including soil and surface waters. This is due to their much slower degradation in the environment and accumulation in soils and sludge (Lee 2010; Oleszczuk 2006, 2007). Fifteen PAHs have been recognised as clearly mutagenic and carcinogenic by the Scientific Committee on Food, of which benzo[a]pyrene and dibenzo[a,h]anthracene are reported to be the most carcinogenic (Scientific Committee on Food 2002; Corredera et al. 2011). In the European Union, the existing law recommends using the PAH4 sum (benzo[a]anthracene, chrysene, benzo[b]fluoranthene and benzo[a]pyrene) as a PAH marker in food (Commission Regulation (EU) 2011).

5-Hydroxymethylfurfural (HMF) is a cyclic aldehyde produced by sugar degradation through the Maillard reaction (a nonenzymatic browning reaction) during food processing or long-term storage of honey (Markowicz et al. 2012). The presence of simple sugars (glucose and fructose) and the many acids and minerals in honey can further enhance the production of this substance (Kuster 1990). HMF concentration is widely recognized as a parameter indicating honey freshness because it is typically absent (or is present in only very small amounts) in fresh honey. Previous studies have reported that honey stored at low temperatures and/or under favourable conditions has low or minimal HMF concentrations, while aged honey and/or honey stored at comparatively higher or medium temperatures has high HMF concentrations (Fallico et al. 2004; Khalil et al. 2010; Shapla et al. 2018). HMF can exert detrimental genotoxic and mutagenic activity through metabolic activation to 5-sulfooxymethylfurfural, and it causes DNA adducts in human beings (Portillo Perez et al. 2019). Honey available for sale may contain no more than 40 mg/kg, except for baker’s and tropical honeys (no more than 80 mg/kg); (Council Directive 2001).

Monitoring contaminant residues in honey helps avoid risks to human health as it is a natural product widely consumed in all population groups, including the most vulnerable, namely children and the elderly (Panseri et al. 2020). Therefore, the main objective of the study was to evaluate the quality of selected European honey in terms of the presence of pesticide residues, PAH levels, and HMF content. Species of honey and origins were taken into account when the obtained results were analysed.

## Materials and Methods

### Sample Collection

The analysed honey samples originated from the retail markets of seven EU countries (Eastern Europe–Poland and Slovakia; Southern Europe–Italy, France and Spain; Northern Europe–Scotland and England). In the case of the Polish samples, the honey was purchased from an industrial region of Poland (Malopolska) as well as a non-industrial one (Warmia and Mazury), the so-called “green lungs of Poland”. The sixteen types of honey collected for the study were as follows: heather, clover, wildflower, multiflorous, linden, rape, buckwheat, forest, honeydew, lemon and orange blossom, thyme, eucalyptus, chestnut, acacia and lavender. The honeys selected for the research were the most representative for each region as they are the most popular and the most purchased.

### Chemicals and Reagents

EPA 525 PAH Mix-B, anthracene d_10_ (IS_1_), chrysene d_12_ (IS_2_), triphenylphosphine (IS_3_), thiametoxam, clothianidine, EPA 531.1 Carbamate Mix, potassium hexacyanoferrate (C_6_FeK_4_N_6_) and zinc acetate dehydrate (C_4_H_6_O_4_Zn*2H_2_O) were obtained from Sigma-Aldrich Chemie GmbH, Germany, and Sant Luis, Missouri, USA. Pesticide-Mix 235, Pesticide-Mix 114 and Pirimicarb were purchased from LGC Standards, UK. Magnesium sulphate anhydrous p.a. and sodium chloride p.a. were from POCh SA, Poland. Acetonitrile, methanol hexane, and glacial formic acid HPLC grade for LiChrosolve® liquid chromatography were purchased from Merck KGaA, Germany. PSA (primary and secondary amine) and C18 SPE Bulk Sorbents were from Agilent Technologies, USA. Deionised water (18MΩ) was produced by a Milli-Q system (Millipore, USA). Stock, intermediate and working-standard solutions of PAHs and pesticides (1 and 100 µg mL^−1^) were prepared in hexane and acetonitrile, respectively.

### Sample Preparation Method

#### Extraction and Clean-up of Samples for Determination of PAHs and Pesticides

The extraction process was based on a modified QuEChERS method that was previously optimised and in-house validated (Surma et al. 2014a, b (A); Surma et al. 2014a, b (B); Surma et al. 2018). In brief, 1.5 g of a representative portion of honey was weighed into a 50 mL centrifuge tube and spiked with all internal standards; this was then mixed and left to stand for 15 min at room temperature prior to extraction. Then, 15 mL of acetonitrile was added, and the mixture was vigorously shaken for 1 min. Next, 1 g of NaCl and 4 g of MgSO_4_ were added; then, the tube was shaken vigorously for another 1 min and centrifuged for 15 min at 8700 RCF. 9 mL of the supernatant was transferred into a PP 15 mL tube containing 230 mg of PSA, 450 mg of C_18_, and 1.200 g of MgSO_4_. After 30 s of shaking and 5 min of centrifugation at 5,000 RCF, 6 mL of the extract was divided into three 2 mL portions, each of which was transferred to a 4 ml tube and evaporated under an N_2_ stream to dryness.

*PAH analysis* The residues (of 2 mL supernatant after evaporation) were dissolved in 0.25 mL of hexane, and the mixture was transferred into an autosampler vial; 1 μL of the extract was then analysed with GC-SIM-MS.

*Pesticide analysis* The residues (of 2 mL supernatant after evaporation) were dissolved in 0.5 mL of acetonitrile; the mixture was then transferred into an autosampler vial, and 1 μL of the extract was analysed with GC-SIM-MS.

#### HMF Analysis

Honey samples were prepared according to Kowalski et al. (2013). In brief, honey samples (about 2.5 g) were dissolved in 10 ml of water and transferred quantitatively to a 25 mL volumetric flask. Then, 0.25 mL of Carrez solution I and 0.25 mL of Carrez solution II were added. The volumetric flask was filled to the mark with deionised water. Before chromatographic analysis, samples were filtered through a 0.45 µm disc filter.

Reagent blank samples were prepared according to the appropriate procedure for all tested analytes. Each sample (real and blank) was prepared in triplicate.

### Instrumentation

The GC application was carried out on a Varian 4000 GC/MS (Varian, Inc., USA) system consisting of a 3800 gas chromatograph and a 4000 Ion Trap MS detector. The column was a DB-5MS column (30 m × 0.25 mm × 0.25 μm; Agilent Technologies, USA). The GC oven was operated with the following temperature programme: initial temperature 50 ºC (1 min)–15 ºC/min–300 ºC (6.0 min) for PAHs; 70 ºC (3 min)–30 ºC/min–150 ºC (1 min)–10 ºC/min–280 ºC (5 min) for pesticides. Helium 5.0 (Linde Gas, Poland) was used as the GC carrier gas at a flow rate of 1.0 mL/min. The autosampling injector was a CP-1177 Split/Splitless Capillary Injector with a temperature of 270 ºC for both analyses and a volume of 1.0 µL; the splitless time was 1.0 min for all standards and samples. Each injection was repeated three times. The ion trap mass spectrometer was operated in internal ionisation mode, and ions were scanned from m/z 45 to 500. An analysis was conducted in the selected ion monitoring mode (SIM), based on the quantitative ions. Analysed compounds were identified according to their qualitative ions and retention times, as summarized in Table S1a (see Supplementary Material – SM). The trap and the transfer line temperatures were set at 180 and 230 °C, respectively, for all tested analytes. The analyses were carried out with a solvent delay of 5 min. The emission current of the ionisation filament was set at 15 µA. Data acquisition and processing were performed using Varian Start Workstation software and NIST 2.0 library (National Institute of Standards and Technology, Gaithersburg, Maryland, USA). An MS1 Minishaker (IKA, Königswinter, Germany) and an MPW 350 R Centrifuge (MPW Med. Instruments, Warsaw, Poland) were employed during the sample preparation. Accublock™ (Labnet, Edison, NJ, USA) with nitrogen 5.0 (Linde Gas, Munich, Germany) was used to evaporate the solvent and to incubate and concentrate the extracts.

The analytes were identified by comparing the retention time and quantitative and qualitative ions using the NIST library. A calibration curve was constructed by plotting the ratio of the peak area, divided by the peak area of the suitable internal standard, against the concentration of the analyte.

HMF qualitative and quantitative analyses were carried out using HPLC–UV/Vis LaChrom ELITE (Merck, Germany). Measurement parameters were as follows: eluent water/methanol 9:1 (v/v), flow rate 1 mL/min, UV detection at 285 nm, column RP-18 Lichrosphere (250 × 4 mm, 5 µm particle size) (Merck, Germany), sample volume 20 µL.

### Standard Preparation

The 6-point calibration curves (expressed by the equation y = ax) for pesticides, PAHs (range 0–1 µg/mL) and for HMF (range 0–20 µg/mL) were prepared by appropriate dilution of standard PAH, pesticide and HMF stock solutions. Calibration parameters (a-calibration slope; r-correlation coefficient) for all analysed compounds are summarized in Table S1a and S1b (see Supplementary Information (SI)).

### Statistical Analysis Method

The data were subjected to statistical analysis using the t-Student test or a one-way ANOVA, followed by Tukey’s post-hoc test or its non-parametric alternatives, i.e., the Mann–Whitney U test, Welch’s t-test, and the Kruskal–Wallis test (if data did not meet the appropriate assumptions). *p* values < 0.05 were considered significant. All analyses were performed using Statistica 13.0 software (Stat-Soft Inc., Tulsa, OK, USA).

## Results and Discussion

### Pesticide Residues

Approximately 80% of wild plants depend on insect pollination, where bees play a pivotal role (Ben Mukiibi et al. 2021; Metz et al. 2020). Honeybees (*Apis mellifera*) readily fly up to a 4 km radius from their apiary, covering an area of about 50 km^2^, thus making them excellent bioindicators of environmental contamination (Malhat et al. 2015). So far, several researchers have reported various pesticide residues in honey at varying concentrations (Blasco et al. 2003; Rissato et al. 2006, 2007; Erdoǧrul 2007; Blasco et al. 2003; Kujawski and Namiesnik 2011; Kujawski et al. 2012; Barganska et al. 2013; Eissa et al. 2014; Saitta et al. 2017; Ben Mukiibi et al. 2021), thus confirming the need to constantly monitor the presence of pesticide residues in honey to ensure its quality and protect consumer health. To evaluate the toxicological significance of human exposure to the pesticide residues found in honey, it is important to compare estimated daily intake (EDI) with the acceptable daily intakes (ADI) established by the FAO/WHO organization (Eissa et al. 2014).

Honey from various parts of Europe was analysed for the presence of nineteen organochlorine pesticides. Four of them (alpha-chlordane, gamma-chlordane, endosulfane, and heptachlor epoxide) were not detected; the other fifteen are shown in table S2 (see SI). Their amount ranged from 0.03 μg/kg to 4.41 μg/kg; DDD was the most frequently detected compound and its presence was observed in fourteen honey samples, which results from the fact that it is a metabolite of the once widely used DDT (dichlorodiphenyltrichloroethane) pesticide. Its highest content was in the sample of Polish rape M honey (0.69 μg/kg), and the lowest was in Slovakian forest honey (0.20 μg/kg). The least frequently detected compound in the analysed honey samples was delta-hexachlorocyclohexane (δ-HCH), which was present only in English wildflower (0.52 μg/kg) and Slovakian rape honey (0.49 μg/kg). Among the tested honey samples, Slovakian rape honey could be considered the most polluted due to the presence of eight organochlorine pesticides (beta-hexachlorocyclohexane, delta-hexachlorocyclohexane, lindane, methoxychlor, aldrin, endrin, endrin ketone, 4,4'-DDD). In contrast, Italian eucalyptus was the least contaminated honey, with only one (endrin aldehyde) detected compound. In an Italian study, 11 organochlorine pesticides were analysed. In 24 out of 26 honeys, residues ranging from traces to 0.15 mg/kg were found (Roggi et al. 1990). Three of the tested honey samples (Scottish clover, Spanish thyme, Slovakian honeydew) were free from contamination with organochlorine pesticides. Due to the scarce amount of organochlorine pesticides present in the analysed honey samples, this honey can be considered safe for consumption. In Blasco et al.’s (2003) research, honey samples from Spain and Portugal showed residues of 42 different pesticides (organochlorine, organophosphates and carbamates), most of which were organochlorine compounds. Among them, gamma-HCH was detected in 50% of the samples, followed by HCB (32%) and other HCH isomers (alpha-HCH and beta-HCH) in 28 and 26% of the samples, respectively.

The statistical analysis of the results did not show any significant differences (*p* > 0.05) in the content of organochlorine compounds in honey in terms of the region or country of origin.

In research on honey samples from Italy that was conducted by Saitta et al. (2017), the presence of 4.4'-DDD (1.15 μg/kg) and endosulfan (1.42 μg/kg) was detected. However, in this study these compounds were not found in the sample from Italy.

In honey from Turkey, the content of β-hexachlorocyclohexane was 0.52 μg/kg (Erdoǧrul 2007). This is a very small value compared to the 22.82 μg/kg found in rapeseed honey from Malopolska. Such a large discrepancy in the amount of this compound may result from the type of raw material from which the honey was produced.

Kujawski et al. (2012) showed the presence of organochlorine pesticides in honey samples from Poland in an amount that does not pose a threat to human health (below 14 μg/kg for sum of 4,4′-DDT and metabolites, and below 5 μg/kg for aldrin, endrin and lindane).

**Fig. 1:**
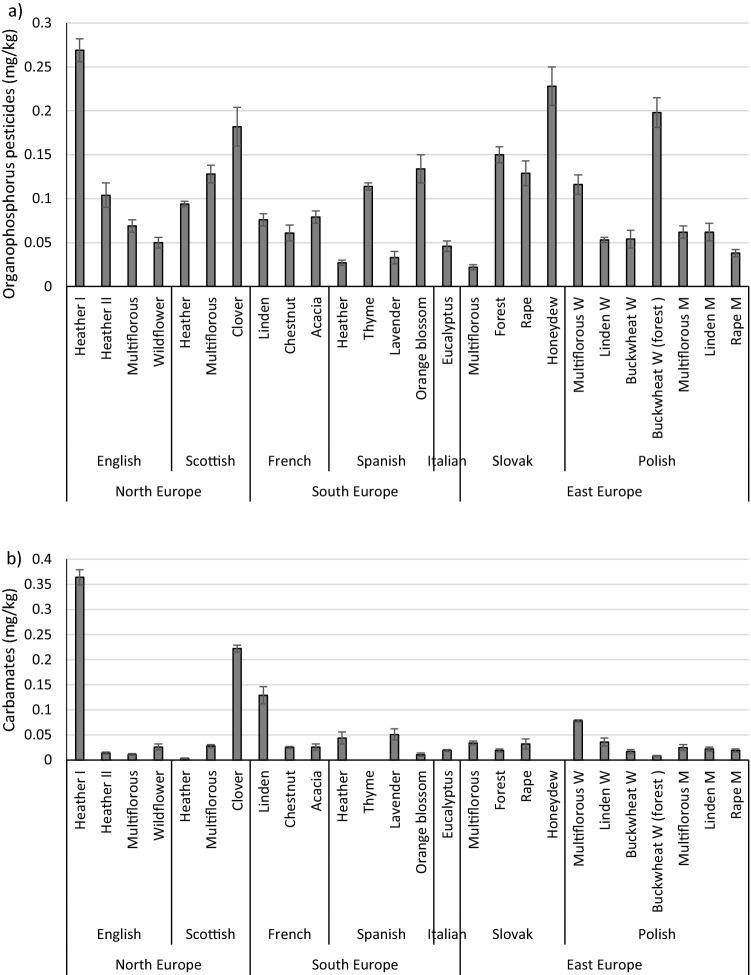

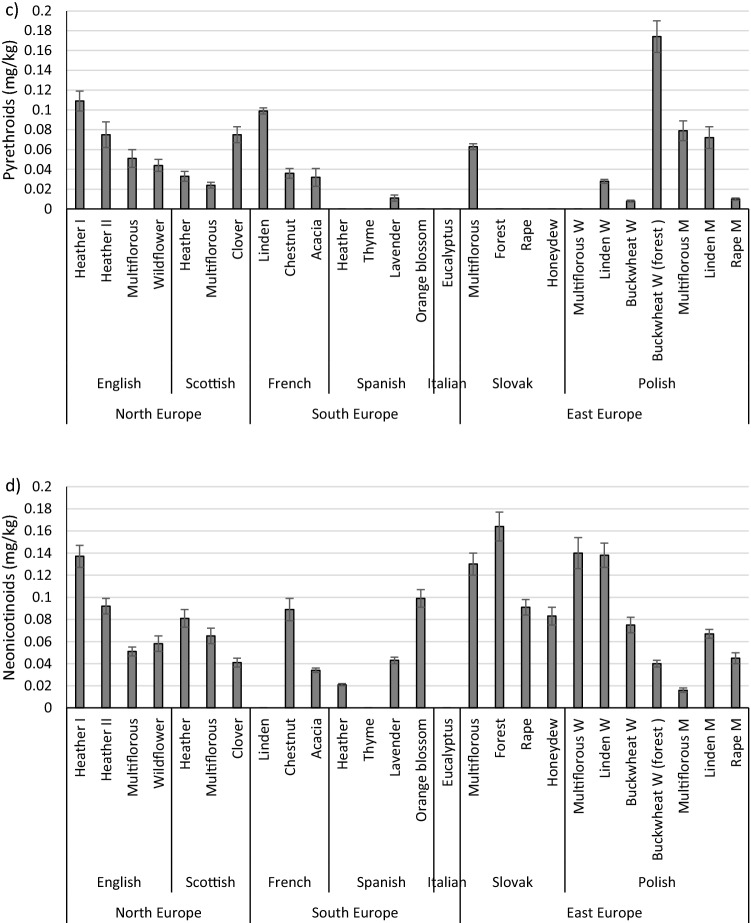
The content of pesticides in honey: **a** Organophosphorus pesticides; **b** Carbamates; **c** Pyrethroids; **d** Neonicotinoids; Values are expressed as means ± standard deviations; *p* values < 0.05; M-honey from the Malopolska region; W-honey from the Warmia and Mazury region

The presence of organophosphorus pesticides was detected in all analysed honey samples (Table 1; Fig. 1a). Diazinon, disulfoton, chlorpyrifos-methyl, parathion-methyl and chlorpyrifos were the most common. At least one detected organophosphorus (OPs) pesticide in each tested honey sample exceeded the acceptable limit. Maximum Residue Limits (MRLs) are established through European Union Regulation (EC) 396/2005 for many pesticides used in agricultural and apiculture practices (Leu and Stenstrom 2010). New MRLs for certain pesticides in honey, ranging from 0.01 to 0.05 mg/kg, have been set since September 2008 by the European Commission (Bargańska et al. 2016). The MRL for the compounds detected in honeys is 0.01 mg/kg; if the pesticide residue level exceeds the MRL, the honey is considered contaminated.

**Table 1:** The content of organophosphorus pesticides in honey (mg/kg)

Region of origin	Country of origin	Type	Diazinon	Disulfoton	Chlorpyrifos-methyl	Parathion-methyl	Malathion	Chlorpyrifos	Parathion ethyl	Ethion
North Europe	England	Heather I	n.d	**0.091 ± 0.007**	n.d	**0.178 ± 0.006**	n.d	n.d	n.d	n.d
		Heather II	**0.017 ± 0.003**	**0.016 ± 0.001**	**0.043 ± 0.004**	**0.028 ± 0.006**	n.d	n.d	n.d	n.d
		Multiflorous	n.d	n.d	**0.047 ± 0.005**	n.d	n.d	**0.022 ± 0.002**	n.d	n.d
		Wildflower	**0.018 ± 0.002**	n.d	n.d	n.d	n.d	**0.032 ± 0.004**	n.d	n.d
	Scotland	Heather	n.d	n.d	**0.09 ± 0.002**	n.d	n.d	0.004 ± 0.001	n.d	n.d
		Multiflorous	n.d	**0.019 ± 0.002**	**0.078 ± 0.004**	**0.024 ± 0.003**	n.d	0.007 ± 0.001	n.d	n.d
		Clover	n.d	**0.025 ± 0.006**	**0.096 ± 0.01**	n.d	n.d	**0.061 ± 0.006**	n.d	n.d
South Europe	France	Linden	**0.016 ± 0.001**	n.d	0.008 ± 0.001	**0.045 ± 0.004**	n.d	0.007 ± 0.001	n.d	n.d
		Chestnut	0.008 ± 0.001	n.d	n.d	n.d	n.d	**0.053 ± 0.008**	n.d	n.d
		Acacia	**0.014 ± 0.002**	n.d	**0.042 ± 0.002**	**0.023 ± 0.003**	n.d	n.d	n.d	n.d
	Spain	Heather	0.006 ± 0.001	**0.014 ± 0.001**	n.d	0.007 ± 0.001	n.d	n.d	n.d	n.d
		Thyme	**0.014 ± 0.003**	n.d	0.1 ± 0.001	n.d	n.d	n.d	n.d	n.d
		Lavender	**0.033 ± 0.007**	n.d	n.d	n.d	n.d	n.d	n.d	n.d
		Orange blossom	**0.019 ± 0.003**	n.d	**0.071 ± 0.008**	n.d	n.d	**0.044 ± 0.005**	n.d	n.d
	Italy	Eucalyptus	**0.019 ± 0.003**	n.d	n.d	n.d	n.d	**0.027 ± 0.003**	n.d	n.d
East Europe	Slovakia	Multiflorous	n.d	n.d	**0.013 ± 0.002**	n.d	n.d	0.009 ± 0.001	n.d	n.d
		Forest	**0.014 ± 0.003**	**0.026 ± 0.002**	**0.11 ± 0.004**	n.d	n.d	n.d	n.d	n.d
		Rape	**0.024 ± 0.003**	n.d	**0.065 ± 0.007**	n.d	n.d	**0.04 ± 0.004**	n.d	n.d
		Honeydew	**0.023 ± 0.002**	**0.021 ± 0.002**	**0.077 ± 0.008**	**0.026 ± 0.001**	n.d	**0.081 ± 0.009**	n.d	n.d
	Poland	Multiflorous W	**0.026 ± 0.004**	n.d	**0.02 ± 0.002**	n.d	n.d	**0.07 ± 0.005**	n.d	n.d
		Linden W	n.d	n.d	**0.053 ± 0.003**	n.d	n.d	n.d	n.d	n.d
		Buckwheat W	n.d	**0.02 ± 0.005**	n.d	**0.025 ± 0.004**	n.d	0.009 ± 0.001	n.d	n.d
		Buckwheat (forest) W	**0.019 ± 0.002**	**0.033 ± 0.002**	**0.038 ± 0.003**	**0.018 ± 0.001**	n.d	**0.09 ± 0.009**	n.d	n.d
		Multiflorous M	n.d	n.d	**0.05 ± 0.005**	n.d	n.d	**0.012 ± 0.002**	n.d	n.d
		Linden M	**0.016 ± 0.002**	**0.023 ± 0.005**	n.d	**0.023 ± 0.003**	n.d	n.d	n.d	n.d
		Rape M	n.d	n.d	**0.018 ± 0.002**	n.d	n.d	**0.02 ± 0.002**	n.d	n.d

Values are expressed as means ± standard deviations; n.d.–not detected; M-honey from the Malopolska region; W-honey from the Warmia and Mazury region; In bold-shaded cells, the Maximum Residue Levels (MRLs) have been exceeded

Chlorpyrifos is one of the most used pesticides in the world for the control of agricultural and non-agricultural pests. According to the World Health Organization, chlorpyrifos is reported to be moderately toxic to humans as it causes autoimmune disorders in the foetus or children and can cause trans-generational mental health effects. Chlorpyrifos was present in 17 of the 26 analysed honey samples, and in 12 of the samples it exceeded the permissible limit (0.01 mg/kg) as its concentration ranged from 0.02 mg/kg to 0.09 mg/kg. The following honey samples were free from chlorpyrifos: English heather I, English heather II, French acacia, Spanish heather, Spanish thyme, Spanish lavender, Slovakian forest, Polish linden W, Polish linden M. The most contaminated honey was Polish buckwheat W (0.09 mg/kg), and the lowest detected content of chlorpyrifos was found in Scottish heather honey (0.004 mg/kg). In a study by Villalba et al. (2020), the highest concentration of chlorpyrifos was found in almost all honey samples from a soybean field (Argentina). These results revealed that land uses and seasonal variations directly impact levels of agrochemicals. The presence of chlorpyrifos-methyl was detected in 18 of the 26 analysed honey samples, where the concentration of this compound ranged from 0.008 to 0.11 mg/kg. The limit of 0.01 mg/kg was exceeded in 16 honey samples (0.02–0.11 mg/kg). The highest level of chlorpyrifos-methyl was observed in Slovakian forest honey (0.11 mg/kg), while the lowest was in Polish multiflorous W honey (0.02 mg/kg). This compound was not detected in English heather I, English wildflower, French chestnut, Spanish heather, Italian eucalyptus, Polish buckwheat W, and Polish linden M. Organophosphorus pesticides such as diazinone, disulfoton, parathion-methyl were present in about 50% of the samples, in most cases exceeding the maximum levels (MLs). The most contaminated honeys were English Heather II, Slovakian Honeydew, and Polish Buckwheat (forest) W. The tested honeys were completely free from malathion, parathion ethyl and ethion (Table 1).

The presence of carbamate pesticides (Fig. 1b), such as oxamyl, propoxur, carbofuran, carbaryl, and methiocarb, was detected in 24 of the 26 analysed honey samples. However, the MRLs of these compounds were not exceeded in more than 90% of the samples. The sum of carbamate insecticides ranged from 0.003  to 0.364 mg/kg. No residue of these pesticides was detected in only two honey samples (Spanish thyme and Slovakian honeydew), whereas English heather I had the highest total carbamate content (0.364 mg/kg), (Fig. 1b). A relatively high concentration of these compounds was also found in three honey samples, ranging from 0.078 to 0.222 mg/kg. In the remaining 19 honey samples, the total amount of carbamate compounds ranged from 0.003 to 0.051 mg/kg (Table 2). The permissible pesticide residue content for oxamyl, carbofuran, carbaryl, methiocarb is established by the EU regulations at the level of 0.05 mg/kg. However, the EU Commission regulation does not include the permitted dose of propoxur in honey. Among all tested honey samples, four of the permissible limits for individual carbamate compounds were exceeded: French linden (carbofuran 0.054 mg/kg), Polish multiflorous W (methiocarb 0.078 mg/kg), Scottish clover (oxamyl 0.195 mg/kg), and English heather I (carbofuran 0.303 mg/kg); (Table 2). In the most contaminated honey sample, the content of carbofuran was 0.303 mg/kg, which is 6.1 times higher than the maximum permissible level of this pesticide residue in honey. Carbaryl, which was detected in a few honey samples, did not exceed the recommended level. The highest number of different carbamate insecticides was observed in French linden honey; however, these values ​​did not exceed the recommended levels.

**Table 2:** The content of carbamate pesticides in honey (mg/kg)

Region of origin	Country of origin	Type	Oxamyl	Propoxur	Carbofuran	Carbaryl	Methiocarb
North Europe	England	Heather I	n.d	0.034 ± 0.007	**0.303 ± 0.005**	0.027 ± 0.003	n.d
		Heather II	n.d	n.d	n.d	n.d	0.014 ± 0.002
		Multiflorous	n.d	0.011 ± 0.002	n.d	n.d	n.d
		Wildflower	n.d	n.d	n.d	0.012 ± 0.003	0.014 ± 0.003
	Scotland	Heather	n.d	0.003 ± 0.001	n.d	n.d	nd
		Multiflorous	n.d	n.d	n.d	n.d	0.028 ± 0.003
		Clover	**0.195 ± 0.001**	n.d	n.d	0.027 ± 0.006	n.d
South Europe	France	Linden	0.022 ± 0.005	0.032 ± 0.003	**0.054 ± 0.004**	0.021 ± 0.005	n.d
		Chestnut	0.009 ± 0.001	0.016 ± 0.001	n.d	n.d	n.d
		Acacia	0.008 ± 0.002	n.d	n.d	0.018 ± 0.004	n.d
	Spain	Heather	n.d	n.d	n.d	0.026 ± 0.006	0.018 ± 0.006
		Thyme	n.d	n.d	n.d	n.d	n.d
		Lavender	n.d	0.03 ± 0.006	0.021 ± 0.005	n.d	n.d
		Orange blossom	n.d	0.011 ± 0.003	n.d	n.d	n.d
	Italy	Eucalyptus	n.d	n.d	n.d	0.019 ± 0.002	n.d
East Europe	Slovakia	Multiflorous	n.d	0.014 ± 0.003	n.d	0.02 ± 0.001	n.d
		Forest	n.d	0.007 ± 0.002	n.d	n.d	0.012 ± 0.001
		Rape	n.d	n.d	n.d	0.017 ± 0.004	0.015 ± 0.006
		Honeydew	n.d	n.d	n.d	n.d	n.d
	Poland	Multiflorous W	n.d	n.d	n.d	nd	**0.078 ± 0.002**
		Linden W	n.d	0.005 ± 0.002	n.d	0.018 ± 0.004	0.013 ± 0.002
		Buckwheat W	n.d	n.d	0.017 ± 0.004	n.d	n.d
		Buckwheat (forest) W	n.d	0.008 ± 0.001	n.d	n.d	n.d
		Multiflorous M	n.d	n.d	n.d	n.d	0.025 ± 0.006
		Linden M	n.d	0.006 ± 0.002	0.016 ± 0.002	n.d	n.d
		Rape M	n.d	0.008 ± 0.002	0.011 ± 0.001	n.d	n.d

Values are expressed as means ± standard deviations; n.d. – not detected; M-honey from the Malopolska region; W-honey from the Warmia and Mazury region; In bold-shaded cells, the Maximum Residue Levels (MRLs) have been exceeded

The presence of pyrethroids such as cyfluthrine, cypermethrine, and flumethrine was detected in 18 of the analysed 26 honey samples (Table 3). The total content of pyrethroid insecticides in the analysed samples ranged from 0.008  to 0.174 mg/kg. No pyrethroid residue was detected in eight samples, mainly from Spain, Italy and Slovakia, while honey samples from the UK were the most contaminated (Fig. 1c). In six samples, all three pyrethroid compounds (cyfluthrine, cypermethrine, flumethrine; Table 3) were detected, but these values ​​did not exceed the limits specified in Regulation (EC) 396/2005 (Commission Regulation (EU) 2005), which defines MRLs for cyfluthrin (0.05 mg/kg) and cypermethrin (0.05–0.2 mg/kg), but not for flumethrin. In 18 honey samples, the content of pyrethroid compounds ranged from 0.005 to 0.091 mg/kg. Only three honey samples exceeded the recommended limit of one of the pyrethroid compounds, namely cyfluthrine (Table 3). An excess of this compound was reported in English heather I (0.062 mg/kg), French linden (0.063 mg/kg), and Polish buckwheat (forest) W (0.091 mg/kg). In the other honey samples, the content of pyrethroid compounds did not exceed the permissible amount (Table 3).

**Table 3:** The content of pyrethroids in honey (mg/kg)

Region of origin	Country of origin	Type	Cyfluthrine	Cypermethrine	Flumethrine
North Europe	England	Heather I	**0.062 ± 0.004**	n.d	0.047 ± 0.006
		Heather II	0.011 ± 0.002	0.049 ± 0.008	0.015 ± 0.003
		Multiflorous	0.031 ± 0.007	0.008 ± 0.001	0.012 ± 0.001
		Wildflower	0.031 ± 0.004	n.d	0.013 ± 0.002
	Scotland	Heather	0.005 ± 0.002	0.012 ± 0.001	0.016 ± 0.002
		Multiflorous	0.011 ± 0.001	0.013 ± 0.002	n.d
		Clover	n.d	n.d	0.075 ± 0.008
South Europe	France	Linden	**0.063 ± 0.001**	n.d	0.036 ± 0.002
		Chestnut	0.009 ± 0.002	n.d	0.027 ± 0.003
		Acacia	0.025 ± 0.004	n.d	0.017 ± 0.005
	Spain	Heather	n.d	n.d	n.d
		Thyme	n.d	n.d	n.d
		Lavender	0.011 ± 0.003	n.d	n.d
		Orange blossom	n.d	n.d	n.d
	Italy	Eucalyptus	n.d	n.d	n.d
East Europe	Slovakia	Multiflorous	0.018 ± 0.001	0.045 ± 0.002	n.d
		Forest	n.d	n.d	n.d
		Rape	n.d	n.d	n.d
		Honeydew	n.d	n.d	n.d
	Poland	Multiflorous W	n.d	n.d	n.d
		Linden W	0.008 ± 0.001	n.d	0.02 ± 0.001
		Buckwheat W	n.d	0.008 ± 0.001	n.d
		Buckwheat (forest) W	**0.091 ± 0.003**	0.033 ± 0.007	0.05 ± 0.006
		Multiflorous M	0.007 ± 0.002	0.035 ± 0.002	0.037 ± 0.006
		Linden M	0.013 ± 0.001	0.041 ± 0.006	0.018 ± 0.004
		Rape M	0.01 ± 0.001	n.d	n.d

Values are expressed as means ± standard deviations; n.d.-not detected; M-honey from the Malopolska region; W-honey from the Warmia and Mazury region; In bold-shaded cells, the Maximum Residue Levels (MRLs) have been exceeded

The presence of clothianidin and thiamethoxam was detected in the range 0.028–0.137 mg/kg and 0.016–0.102 mg/kg, respectively (Table 4). The Commission Regulation (EU) 2017/671 of 7 April 2017 (Commission Regulation (EU) 2017) specifies the Maximum Residue Limits (MRLs) of 0.05 mg/kg for clothianidin and thiamethoxam in honey and other apiculture products. Clothianidin was found in 15 out of the 26 tested samples; in 9 of them, its level exceeded the maximum permissible limit. The highest levels were found in heather honey from the UK (0.051–0.137 mg/kg); the least contaminated honeys (< 0.05 mg/kg) were from Scotland, France and Poland (the Malopolska region). In studies conducted by Woodcock et al. (2018), clothianidin was the most frequently detected neonicotinoid in honey samples from the UK, but its concentration was low (< 2.0 ng/g). The presence of thiamethoxam was detected in 15 samples, but the level exceeded the MRL in only 8 of them. Honey from Italy, Spain and France did not exceed the permissible level of thiamethoxam residue, while honey from Slovakia was the most polluted with this residue (0.043–0.102 mg/kg). Although this pesticide is commonly used to treat rapeseed, it was not detected in the analysed rapeseed honey (Table 4). Of all the tested honey samples, only 3 of them were completely free of neonicotinoid compounds: French linden, Spanish thyme and Italian eucalyptus (Fig. 1d).

**Table 4:** The content of neonicotinoids in honey (mg/kg)

Region of origin	Country of origin	Type	Clothianidin	Thiamethoxam
North Europe	England	Heather I	**0.137 ± 0.01**	n.d
		Heather II	**0.092 ± 0.007**	n.d
		Multiflorous	**0.051 ± 0.004**	n.d
		Wildflower	n.d	**0.058 ± 0.007**
	Scotland	Heather	n.d	**0.081 ± 0.008**
		Multiflorous	n.d	**0.065 ± 0.007**
		Clover	n.d	0.041 ± 0.004
South Europe	France	Linden	n.d	n.d
		Chestnut	0.042 ± 0.005	0.047 ± 0.005
		Acacia	0.034 ± 0.002	nd
	Spain	Heather	n.d	0.021 ± 0.001
		Thyme	n.d	n.d
		Lavender	0.043 ± 0.003	n.d
		Orange blossom	**0.075 ± 0.006**	0.024 ± 0.002
	Italy	Eucalyptus	n.d	n.d
East Europe	Slovakia	Multiflorous	0.028 ± 0.003	**0.102 ± 0.007**
		Forest	**0.08 ± 0.006**	**0.084 ± 0.007**
		Rape	**0.091 ± 0.007**	n.d
		Honeydew	0.04 ± 0.003	0.043 ± 0.005
	Poland	Multiflorous W	**0.08 ± 0.008**	0.06 ± 0.006
		Linden W	**0.067 ± 0.005**	**0.071 ± 0.006**
		Buckwheat W	**0.075 ± 0.007**	n.d
		Buckwheat (forest) W	n.d	0.04 ± 0.003
		Multiflorous M	n.d	0.016 ± 0.002
		Linden M	n.d	**0.067 ± 0.004**
		Rape M	0.045 ± 0.005	n.d

Values are expressed as means ± standard deviations; n.d. – not detected; M-honey from the Malopolska region; W-honey from the Warmia and Mazury region; In bold-shaded cells, the Maximum Residue Levels (MRLs) have been exceeded.

In the analysed honey samples, no significant (*p* > 0.05) differences were found between the concentration of individual organophosphorus, carbamate, pyrethroid or neonicotinoid pesticides and the country or region of origin (data not shown).

In studies by Ponce-Vejar et al. (2022), the pesticides the most frequently found at higher concentrations were neonicotinoids, followed by organophosphates, herbicides, and fungicides. The number, frequency, and concentration of pesticides were higher in samples collected from hives located where intensive and highly technified agriculture is practiced. These honey samples originated from the state of Jalisco, which is the most productive agricultural state in Mexico.

### Polycyclic Aromatic Hydrocarbons

**Fig. 2:**
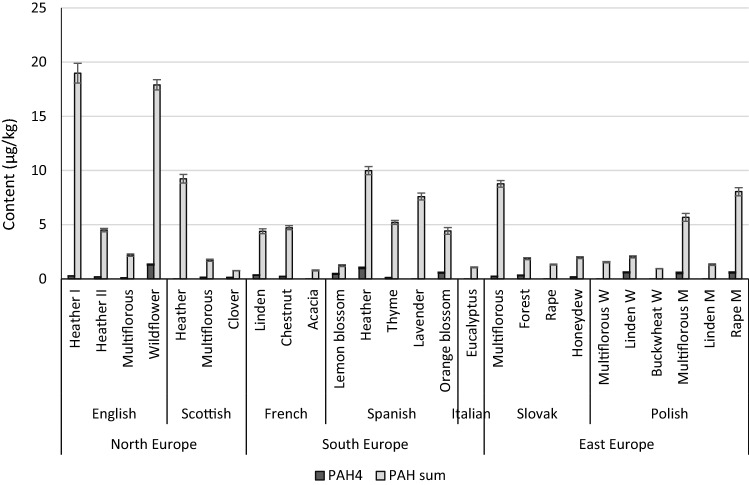
The levels of the PAH4 and PAH sum in honey; Values are expressed as means ± standard deviation calculated from uncertainty propagation law; *p* values < 0.05

Polycyclic aromatic hydrocarbons were detected in most of the analysed honey samples (Table S3; see SI). Four of the most dangerous compounds were not detected in any of the examined samples: benzo[k]fluoranthene, indeno[c,d]pyrene, dibenzo[a,h]anthracene and benzo[g,h,i]perylene. The total sum of PAHs was in the range 0.76 to 18.98 μg/kg. The PAH4 content in the honey samples ranged from 0.1 to 1.32 μg/kg (Fig. 2). The mean concentration of these PAHs was 0.28 µg/kg. English wildflower honey was the most contaminated with PAHs: chrysene (0.77 µg/kg) and benzo[a]anthracene (0.55 µg/kg) were detected. The least contaminated was English multi-flower honey, in which benzo[b]fluoranthene was detected at the level of 0.1 µg/kg. Among all the tested honey samples, the presence of benzo[a]pyrene was detected only in Slovakian forest (0.32 µg/kg) and Polish linden W honey (0.50 µg/kg); (Table S3; see SI).

There were significant differences (*p* < 0.05) in the amount of acenapthylene and acenaphthene in the tested honey samples, depending on the region of origin. The acenapthylene concentration was significantly higher in honey samples from North Europe than those from East Europe. However, in the honey from North Europe, the amount of acenaphthene was significantly lower than in the honey from South Europe. There were no significant differences (*p* > 0.05) in PAH concentrations between countries in the conducted research (data not shown).

Commission Regulation (EU) No. 835/2011 of August 19 Regulation (EC) No. 1881/2006 (Commission Regulation (EU) 2011) sets maximum levels for polycyclic aromatic hydrocarbons (PAH4) in oils, fats, smoked meats, smoked fish and sea food, processed cereal-based food, baby food, infant’s formula, and milk and foods for special medical purposes for infants. There are no defined maximum levels for PAH4 in honey. According to the Commission Regulation (EU) 2015/1933 of 27 October 2015 (Commission Regulation (EU) 2015), the maximum content of benzo(a)pyrene, which is used as a marker for the occurrence and effect of carcinogenic polycyclic aromatic hydrocarbons in food supplements containing royal jelly, should not exceed 10 μg/kg. The levels of PAH4 in the investigated samples were lower than these established limits; therefore, it can be concluded that the tested honeys are safe products that do not pose any risk to consumers. However, the obtained results emphasise the need for further research in this area and the necessity to set maximum PAH levels for honey to minimise the risk for human health.

### 5-Hydroxymethylfurfural

**Fig. 3:**
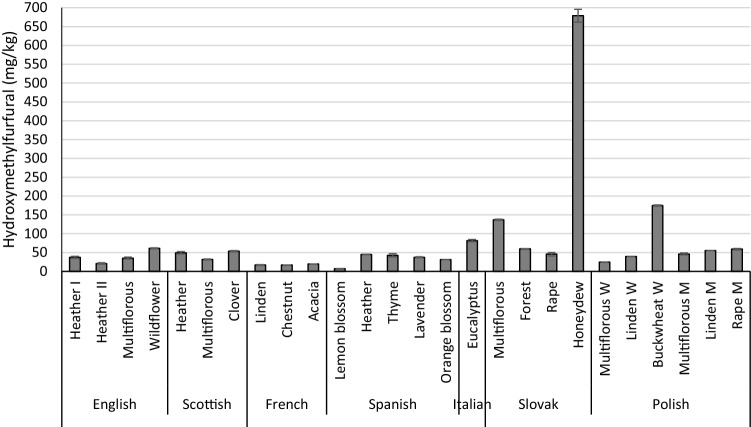
The 5-hydroxymethylfurfural (HMF) content in the investigated honey; Values are expressed as means ± standard deviations; *p* values < 0.05

Honey available for sale may contain no more than 40 mg/kg of HMF, except for baker’s and tropical honeys (the norm: no more than 80 mg/kg) (Council Directive 2001). HMF was detected in all tested honey samples, and 45% of the samples exceeded the recommended level. The content of hydroxymethylfurfural (HMF) for individual types of honey ranged from 7.29 to 678.77 mg/kg (Fig. 3); the lowest value was determined in Spanish lemon blossom honey; the highest was in Slovakian honeydew, in which the HMF standards were exceeded 17 times. Only samples of French honey (16.93–19.63 mg/kg) met the acceptable standard for HMF content. Honey samples from Spain in most cases did not exceed the HMF limits, and their content ranged from 7.29 mg/kg to 44.91 mg/kg. Honey of English, Scottish, Slovak, Italian and Polish origin exceeded the permissible level of HMF, determined by Council Directive 2001/110/EC of 20 December 2001 (Council Directive 2001).

There were significant (*p* < 0.05) differences in the amount of HMF content between countries and regions. Honey samples from Slovakia were the most contaminated with HMF, while those from France were least contaminated. Consequently, honey from East Europe was characterized by a higher HMF content than honey from South Europe. The work revealed the relation between HMF content and honeys of different origins; in contrast, no such influence was found either in the case of pesticides or PAHs. It was observed that the HMF level was lowest and highest in the lightest-coloured honey and dark honey, respectively.

Zappalà et al. (2005) determined the HMF content in acacia, lemon blossom, eucalyptus, chestnut and wildflower honey using HPLC. Slightly lower values ​​were obtained for chestnut (n.d.–4.1 mg/kg) and acacia honey (8.4–16.2 mg/kg), and higher values were found for lemon honey (8.1–45.2 mg/kg). HMF was not present in eucalyptus honey. The highest levels were detected for wildflower honey (85.5 mg/kg).

HMF content was determined by Apriceno et al. (2018) in wildflower, acacia, orange blossom, forest, chestnut, eucalyptus, lemon blossom, honeydew and thyme honey: the lowest levels of HMF were determined in eucalyptus honey (8.68–25.69 mg/kg), forest (11.05 mg/kg), honeydew (6.05–24.78 mg/kg) and thyme honey (26.71 mg/kg). The highest values ​​were recorded for lemon blossom (38.84 mg/kg), orange blossom (54.47 mg/kg) and chestnut (18.67–87.37 mg/kg). Also, similar HMF levels were observed in wildflower (15.06–82.63 mg/kg) and acacia honey (2.31–103 mg/kg).

Popek et al. (2017) found lower levels of HMF in linden honey (0.95 mg/kg), buckwheat honey (1.72 mg/kg) and rape (0.86 mg/kg). Pasias et al. (2017) also determined the HMF content in lemon blossom as well as in multiflorous and heather honey. In the case of heather honey, they noted similar values ​​(7.1 mg/kg and 38 mg/kg). Both lower and higher values were found for lemon blossom (2.5 mg/kg and 26 mg/kg), as compared to our results (7.29 mg/kg). Lower results were obtained from multiflorous honey (2.4–22 mg/kg).

HMF contamination of lavender honey was investigated by Żak et al. (2017), who obtained lower values ​​(2.04 mg/kg). The same results were also achieved by Popek et al. (2017), who determined HMF in linden (0.95 mg/kg), buckwheat (1.72 mg/kg) and rape honey (0.86 mg/kg).

Based on the literature and data, it can be concluded that the results are inconsistent. The analysis focused only on a single batch from each producer, which makes it impossible to identify the cause of these discrepancies.

## Conclusion

Honey is synonymous with healthy food. However, its quality should be taken into account, as should, above all, the health risks of contamination in honey because its quality is related, among other things, to the state of the environment. The constant exposure of bees to various types of chemicals affects the honey they produce. Therefore, knowing the degree of honey contamination can be of great importance to human health. In this study, twenty-six selected honey samples from different species and countries of origin were assessed for pesticide residue content, PAH levels and HMF levels. In general, the obtained results showed that most of the analysed compounds were present in the tested honey samples. In addition, the detected organophosphorus pesticides, neonicotinoids and HMF exceeded the recommended maximum levels (MLs) in most of the samples. It can be assumed that the main source of contamination of the tested honeys is commonly used agrochemicals. Therefore, the use of pesticides and other agricultural chemicals should be limited. We also need to accelerate the transition to less intensive, more sustainable farming methods.

## Supplementary Information

Below is the link to the electronic supplementary material.

Supplementary file1 (DOCX 62 kb)
