# Supplementary material for: Levels of Contamination by Pesticide Residues, Polycyclic Aromatic Hydrocarbons (PAHs), and 5-Hydroxymethylfurfural (HMF) in Honeys Retailed in Europe
Source: Arch Environ Contam Toxicol. 2023 Jan 2;84(2):165–78. doi: 10.1007/s00244-022-00970-3 (PMC9968705; doi:10.1007/s00244-022-00970-3)
Supplement: Supplementary file 1 — Supplementary file1 (DOCX 62 kb) [file 244_2022_970_MOESM1_ESM.docx]

Supplementary Information (SI)

Supplementary Table S1a, Table S1b, Table S2, Table S3

**Table S1a** Parameters of GC-MS analysis and calibration of examined compounds

| R_t_ [min] | Compound | Quantification ion | Confirmation ions | a | r |
| --- | --- | --- | --- | --- | --- |
| PAHs | | | | | |
| 7.43 | NaP | 128.3 | 128.2, 102.2, 127.5 | 0.0050 | 0.9973 |
| 8.50 | MeNaP2 | 142.3 | 142.2, 141.4, 115.3 | 0.0035 | 0.9928 |
| 8.67 | MeNaP1 | 141.4 | 141.2, 142.2, 115.2 | 0.0033 | 0.9969 |
| 9.94 | Acp | 152.1 | 151.1, 151.3, 153.1 | 0.0062 | 0.9958 |
| 10.22 | Ace | 153.3 | 153.2, 154.2, 152.4 | 0.0042 | 0.9975 |
| 11.06 | Flu | 166.1 | 164.1, 165.1, 165.3 | 0.0044 | 0.9937 |
| 12.61 | Phen | 178.1 | 166.1, 178.2, 179.1 | 0.0067 | 0.9945 |
| 12.67 | Ant-d_10_ (IS_1_) | 188.0 | 188.1, 177.9, 189.2 | - | - |
| 12.69 | Ant | 178.1 | 165.1, 178.2, 179.1 | 0.0066 | 0.9997 |
| 14.54 | Fla | 202.5 | 202.4, 200.6, 201.7 | 0.0086 | 0.9993 |
| 14.90 | Pyr | 202.1 | 200.1, 202.3, 203.1 | 0.0096 | 0.9965 |
| 16.85 | B[a]a | 228.1 | 226.1, 228.3, 229.1 | 0.0070 | 0.9971 |
| 16.87 | Chr-d_12_ (IS_2_) | 240.1 | 240.2, 239.2, 241.2 | - | - |
| 16.91 | Chr | 228.1 | 226.1, 228.3, 229.1 | 0.0081 | 0.9992 |
| 18.61 | B[b]f | 252.1 | 250.1, 253.1, 253.3 | 0.0094 | 0.9945 |
| 18.66 | B[k]f | 252.1 | 250.1, 250.4, 253.1 | 0.0088 | 0.9973 |
| 19.23 | B[a]p | 252.1 | 250.1, 250.3, 253.2 | 0.0096 | 0.9947 |
| 21.96 | I[cd]p | 276.1 | 274.1, 277.1, 277.5 | 0.0070 | 0.9975 |
| 22.07 | D[ah]a | 278.2 | 276.0, 276.5, 279.1 | 0.0094 | 0.9959 |
| 22.73 | B[ghi]P | 276.0 | 274.1, 276.4, 277.0 | 0.0115 | 0.9955 |
| Pesticides | | | | | |
| 7.00 | Aldicarb | 57.1 | 57.0, 43.0, 71.1 | 0.0063 | 0.9905 |
| 7.58 | Dichlorvos | 185.0 | 109.0, 185.2, 79.0 | 0.0060 | 0.9953 |
| 10.01 | Mevinphos | 127.2 | 127.0, 109.0, 191.1 | 0.0079 | 0.9978 |
| 10.39 | Oxamyl | 162.0 | 147.1, 149.1, 55.0 | 0.0014 | 0.9988 |
| 10.89 | Clothianidine | 132.0 | 74.1, 51.0, 109.0 | 0.0025 | 0.9965 |
| 12.25 | Propoxur | 110.0 | 110.2, 152.0, 81.0 | 0.0118 | 0.9930 |
| 13.36 | α-HCH | 180.8 | 182.9, 181.1, 218.9 | 0.0309 | 0.9990 |
| 13.56 | Dimethoate | 87.1 | 93.0, 125.0, 92.0 | 0.0021 | 0.9913 |
| 13.75 | Carbofuran | 164.1 | 164.0, 149.0, 131.0 | 0.0013 | 0.9971 |
| 13.88 | β-HCH | 180.8 | 181.0, 182.9, 218.9 | 0.0308 | 0.9943 |
| 14.06 | Lindane | 182.8 | 181.0, 182.9, 218.9 | 0.0319 | 0.9941 |
| 14.28 | Diazinon | 179.2 | 179.1, 137.0, 152.1 | 0.0222 | 0.9850 |
| 14.52 | Disulfoton | 88.1 | 88.0, 89.0, 124.9 | 0.0119 | 0.9985 |
| 14.65 | δ-HCH | 180.8 | 181.1, 182.9, 218.9 | 0.0225 | 0.9981 |
| 14.84 | Pirimicarb | 166.2 | 166.1, 149.0, 72.1 | 0.0356 | 0.9992 |
| 15.38 | Chlorpyrifos-methyl | 286.1 | 286.0, 287.9, 124.9 | 0.0188 | 0.9977 |
| 15.56 | Methyl parathion | 109.0 | 124.9, 109.1, 79.0 | 0.0031 | 0.9945 |
| 15.68 | 3-hydroxycarbofuran | 180.4 | 205.1, 175.1, 91.0 | 0.0044 | 0.9901 |
| 15.84 | Heptachlor | 272.0 | 272.1, 273.9, 270.1 | 0.0206 | 0.9990 |
| 15.95 | Carbaryl | 144.0 | 144.1, 115.1, 271.9 | 0.0140 | 0.9952 |
| 16.21 | Methiocarb | 153.0 | 153.1, 109.1, 91.0 | 0.0002 | 0.9964 |
| 16.44 | Malathion | 125.1 | 125.1, 93.0, 173.0 | 0.0065 | 0.9970 |
| Pesticides | | | | | |
| 16.61 | Chlorpyrifos | 197.0 | 96.9, 197.1, 198.9 | 0.0087 | 0.9967 |
| 16.78 | Aldrin | 263.0 | 263.1, 91.0, 265.0 | 0.0062 | 0.9917 |
| 16.74 | Fenthion | 278.3 | 278.1, 91.0, 79.0 | 0.0268 | 0.9947 |
| 16.85 | Parathion ethyl | 109.1 | 109.0, 96.9, 91.0 | 0.0031 | 0.9969 |
| 17.22 | Bromophos | 331.1 | 207.1, 78.1, 201.2 | 0.0188 | 0.9900 |
| 17.41 | Thiamethoxam | 212.1 | 212.0, 132.0, 247.0 | 0.0029 | 0.9951 |
| 17.82 | Heptachlor epoxide | 352.6 | 353.0, 354.8, 81.1 | 0.0092 | 0.9986 |
| 17.53 | Chlorphenvinphos | 267.2 | 267.0, 269.0, 323.1 | 0.0036 | 0.9933 |
| 18.44 | Bromophos-ethyl | 97.0 | 96.9, 303.1, 358.9 | 0.0137 | 0.9960 |
| 18.53 | γ-chlordane | 373.0 | 373.1, 371.3, 237.0 | 0.0077 | 0.9984 |
| 18.75 | Aldicarb sulfone | 87.1 | 87.0, 143.1, 199.2 | 0.0010 | 0.9935 |
| 18.94 | α-chlordane | 372.6 | 373.0, 236.9, 239.0 | 0.0087 | 0.9953 |
| 19.65 | DDE | 245.8 | 246.1, 248.0, 318.0 | 0.0420 | 0.9966 |
| 19.77 | Dieldrin | 78.9 | 79.1, 81.0, 77.1 | 0.0044 | 0.9966 |
| 20.50 | Endrin | 80.9 | 81.1, 262.9, 243.0 | 0.0067 | 0.9902 |
| 20.89 | Endosulfan | 194.8 | 195.0, 159.1, 240.9 | 0.0035 | 0.9959 |
| 21.21 | DDD | 234.9 | 235.1, 165.0, 237.0 | 0.0333 | 0.9941 |
| 21.14 | Ethion | 97.0 | 235.1, 165.0, 237.0 | 0.0154 | 0.9999 |
| 21.50 | Endrin aldehyde | 245.0 | 344.9, 249.9, 243.1 | 0.0066 | 0.9967 |
| 22.47 | Endosulfan sulfate | 271.7 | 271.9, 237.0, 239.0 | 0.0086 | 0.9908 |
| 22.75 | DDT | 234.9 | 235.1, 237.0, 165.0 | 0.0096 | 0.9920 |
| 23.54 | TPP (IS_3_) | 325.3 | 325.0, 326.1, 77.1 | - | - |
| 24.42 | Endrin ketone | 316.7 | 317.0, 318.9, 315.1 | 0.0070 | 0.9965 |
| 24.87 | bifenthrin | 165.1 | 181.1, 165.0, 267.2 | 0.0031 | 0.9967 |
| 25.06 | Methoxychlor | 226.9 | 227.1, 228.1, 165.0 | 0.0095 | 0.9985 |
| 25.86 | Phenothrin (isomer 1) | 183.1 | 123.1, 183.0, 165.1 | 0.0075 | 0.9971 |
| 26.14 | phenothrin (isomer 2) | 165.2 | 123.1, 183.0, 165.1 | 0.0062 | 0.9946 |
| 27.04 | Mirex (IS_4_) | 271.7 | 272.0, 236.9, 270.1 | - | - |
| 28.83 | permethrin (isomer 1) | 163.0 | 183.0, 165.0, 163.1 | 0.0020 | 0.9988 |
| 29.08 | permethrin (isomer 2) | 163.0 | 183.0, 165.0, 163.1 | 0.0027 | 0.9926 |
| 29.80 | cyfluthrin (isomer 1) | 163.0 | 206.3, 163.1, 165.0 | 0.0026 | 0.9970 |
| 30.34 | cyfluthrin (isomer 2) | 163.0 | 206.3, 163.1, 165.0 | 0.0021 | 0.9962 |
| 30.52 | cypermethrin (isomer 1) | 163.0 | 181.1, 163.1, 165.0 | 0.0029 | 0.9979 |
| 30.58 | cypermethrin (isomer 2) | 163.2 | 181.0, 163.0, 164.9 | 0.0021 | 0.9962 |
| 30.65 | cypermethrin (isomer 2) | 165.1 | 181.0, 163.0, 164.9 | 0.0020 | 0.9984 |
| 31.64 | fenvalerate (isomer 1) | 225.2 | 225.2, 181.0, 226.1 | 0.0031 | 0.9935 |
| 31.94 | fenvalerate (isomer 2) | 181.0 | 181.1, 225.2, 267.2 | 0.0036 | 0.9983 |
| 32.75 | deltamethrin | 181.1 | 181.0, 253.1, 255.2 | 0.0019 | 0.9959 |

R_t_ – retention time; a – calibration slope; r – correlation coefficient; NaP – naphthalene; MeNaP2 – 2-methylnaphthalene; MeNaP1 –1-methylnaphthalene; Ace – acenaphthene; Acp – acenaphthylene; Flu – fluorene; Phen – phenanthrene; Ant-d10 – anthracene-d10; Ant – anthracene; Fla – Fluoranthene; Pyr – pyrene; B[a]a – benzo[a]anthracene; Chr-d12 – chrysene-d12; Chr – chrysene; B[b]f – benzo[b]fluoranthene; B[k]f – benzo[k]fluoranthene; B[a]p – benzo[a]pyrene; I[cd]p – indeno[1,2,3-c,d]pyrene; D[ah]a – dibenzo[a,h]anthracene; B[ghi]P – benzo[g,h,i]perylene; HCH – hexachlorocyclohexane; TPP- triphenylphosphine

**Table S1b** Parameters of HPLC analysis and calibration of examined compound

| R_t_ [min] | Compound | λ | a | r |
| --- | --- | --- | --- | --- |
| 6.92 | HMF | 285 nm | 0.0050 | 0.9973 |

R_t_ – retention time; a – calibration slope; r – correlation coefficient; λ – wavelength

**Table S2** The content of OCPs in honey (mg/kg)

| Region of origin | Country of origin | Type | α-HCH | β-HCH | δ-HCH | Lindane | Heptachlor | Methoxychlor | Aldrin | Dieldrin | Endrin | Endrin aldehyde | Endrin ketone | Endosulfan sulfate | o,p' - DDE | 4,4'-DDD | 4,4' -DDT |
| --- | --- | --- | --- | --- | --- | --- | --- | --- | --- | --- | --- | --- | --- | --- | --- | --- | --- |
| North Europe | England | Heather I | 2.37 ± 0.07 | n.d. | n.d. | 0.68 ± 0.03 | n.d. | n.d. | n.d. | n.d. | 1.62 ±  0.06 | 0.86 ±  0.06 | n.d. | n.d. | n.d. | n.d. | 0.28 ± 0.01 |
|  | England | Heather II | n.d. | n.d. | n.d. | n.d. | n.d. | n.d. | n.d. | n.d. | n.d. | n.d. | n.d. | n.d. | n.d. | 0.34 ± 0.03 | n.d. |
|  | England | Multiflorous | n.d | n.d. | n.d. | n.d. | n.d. | n.d. | 0.45 ± 0.03 | n.d. | 0.72 ±  0.06 | 0.41 ±  0.03 | n.d. | n.d. | n.d. | n.d. | n.d. |
|  | England | Wildflower | 1.17 ± 0.04 | n.d | 0.52 ± 0.02 | n.d. | n.d. | n.d. | n.d. | 0.43 ±  0.02 | n.d. | n.d. | n.d. | n.d. | n.d. | 0.32 ± 0.03 | n.d. |
|  | Scotland | Heather | n.d. | n.d.. | n.d. | 0.14 ± 0.01 | n.d. | n.d. | n.d. | 0.72 ±  0.07 | 1.57 ±  0.06 | n.d. | 2.18 ± 0.12 | n.d. | n.d. | 0.26 ± 0.01 | n.d. |
|  | Scotland | Multiflorous | 0.54 ± 0.05 | 3.97 ± 0.19 | n.d. | 4.07 ± 0.18 | n.d. | n.d. | n.d. | n.d. | n.d. | n.d. | n.d. | n.d. | n.d. | 0.36 ± 0.02 | n.d. |
|  | Scotland | Clover | n.d. | n.d. | n.d. | n.d. | n.d. | n.d. | n.d. | n.d. | n.d. | n.d. | n.d. | n.d. | n.d. | n.d. | n.d. |
| South Europe | France | Linden | n.d. | n.d. | n.d. | n.d. | 2.95 ±  0.11 | n.d. | 0.17 ± 0.01 | 0.70 ±  0.03 | n.d. | n.d. | n.d. | n.d. | 0.04 ±  0.00 | 0.39 ± 0.04 | 0.32 ± 0.01 |
|  | France | Chestnut | n.d. | n.d. | n.d. | n.d. | n.d. | n.d. | n.d. | n.d. | n.d. | n.d. | 1.63 ± 0.12 | 3.16 ±  0.23 | n.d. | 0.26 ± 0.01 | n.d. |
|  | France | Acacia | n.d. | n.d. | n.d. | n.d. | 1.93 ±  0.05 | n.d. | n.d. | n.d. | n.d. | n.d. | n.d. | n.d. | n.d. | n.d. | n.d. |
|  | Spain | Lemon blossom | n.d. | n.d. | n.d. | n.d. | n.d. | n.d. | n.d. | 0.97 ±  0.02 | n.d. | 0.68 ±  0.03 | 1.74 ± 0.05 | 0.75 ±  0.06 | n.d. | n.d. | 0.38 ± 0.02 |
|  | Spain | Heather | n.d. | n.d. | n.d. | n.d. | 4.32 ±  0.13 | n.d. | n.d. | n.d. | n.d. | 1.09 ±  0.06 | 4.41 ± 0.04 | 2.62 ±  0.04 | n.d. | 0.60 ± 0.04 | 0.27 ± 0.01 |
|  | Spain | Thyme | n.d. | n.d. | n.d. | n.d. | n.d. | n.d. | n.d. | n.d. | n.d. | n.d. | n.d. | n.d. | n.d. | n.d. | n.d. |
|  | Spain | Lavender | n.d. | 0.70 ± 0.02 | n.d. | n.d. | n.d. | 0.03 ±  0.00 | n.d. | n.d. | n.d. | n.d. | n.d. | n.d. | n.d. | 0.26 ± 0.01 | 0.25 ± 0.00 |
|  | Spain | Orange blossom | 0.25 ± 0.00 | n.d. | n.d. | n.d. | n.d. | n.d. | 0.36 ± 0.02 | n.d. | n.d. | n.d. | n.d. | 2.32 ±  0.12 | n.d. | 0.49 ± 0.05 | n.d. |
|  | Italy | Eucalyptus | n.d. | n.d. | n.d. | n.d. | n.d. | n.d. | n.d. | n.d. | n.d. | n.d. | 2.73 ± 0.03 | n.d. | n.d. | n.d. | n.d. |
| East Europe | Slovakia | Multiflorous | n.d. | n.d. | n.d. | n.d. | n.d. | n.d. | n.d. | 0.46 ±  0.02 | 0.56 ±  0.03 | n.d. | n.d. | 1.06 ±  0.04 | 0.05 ±  0.00 | 0.59 ± 0.05 | 0.38 ± 0.02 |
|  | Slovakia | Forest | n.d. | 1.06 ± 0.03 | n.d. | n.d. | n.d. | n.d. | n.d. | n.d. | n.d. | n.d. | n.d. | n.d. | n.d. | 0.20 ± 0.02 | n.d. |
|  | Slovakia | Rape | n.d. | 1.89 ± 0.19 | 0.49 ± 0.02 | 2.36 ± 0.01 | n.d. | 0.07 ±  0.00 | 0.57 ± 0.05 | n.d. | 2.22 ±  0.16 | n.d. | 2.02 ± 0.12 | n.d. | n.d. | 0.25 ± 0.02 | n.d. |
|  | Slovakia | Honeydew | n.d. | n.d. | n.d. | n.d. | n.d. | n.d. | n.d. | n.d. | n.d. | n.d. | n.d. | n.d. | n.d. | n.d. | n.d. |

| Region of origin | Country of origin | Type | α-HCH | β-HCH | δ-HCH | Lindane | Heptachlor | Methoxychlor | Aldrin | Dieldrin | Endrin | Endrin aldehyde | Endrin ketone | Endosulfan sulfate | o,p' - DDE | 4,4'-DDD | 4,4' -DDT |
| --- | --- | --- | --- | --- | --- | --- | --- | --- | --- | --- | --- | --- | --- | --- | --- | --- | --- |
|  | Poland | Multiflorous W | 0.50 ± 0.05 | n.d. | n.d. | n.d. | 4.17 ±  0.35 | n.d. | n.d. | n.d. | n.d. | n.d. | n.d. | n.d. | n.d. | n.d. | 0.20 ± 0.01 |
| East Europe | Poland | Linden W | n.d. | n.d. | n.d. | 0.24 ± 0.01 | n.d. | n.d. | n.d. | n.d. | 3.10 ±  0.25 | n.d. | n.d. | n.d. | 0.06 ±  0.00 | n.d. | 0.21 ± 0.00 |
|  | Poland | Buckwheat W | 0.13 ± 0.00 | 1.15 ± 0.08 | n.d. | 1.00 ± 0.06 | 1.04 ±  0.03 | 0.06 ±  0.00 | 1.68 ± 0.07 | n.d. | n.d. | n.d. | n.d. | n.d. | n.d. | n.d. | n.d. |
|  | Poland | Multiflorous M | n.d. | n.d. | n.d. | n.d. | 2.51 ±  0.08 | n.d. | n.d. | 1.12 ±  0.07 | n.d. | n.d. | n.d. | n.d. | n.d. | n.d. | n.d. |
|  | Poland | Linden M | 0.55 ± 0.02 | n.d. | n.d. | n.d. | 3.64 ±  0.13 | 0.11 ±  0.00 | 0.27 ± 0.01 | n.d. | n.d. | n.d. | n.d. | 1.49 ±  0.02 | n.d. | 0.60 ± 0.04 | n.d. |
|  | Poland | Rape M | 0.56 ± 0.06 | 22.82 ± 0.21 | n.d. | n.d. | n.d. | n.d. | n.d. | 0.72 ±  0.03 | n.d. | n.d. | n.d. | n.d. | n.d. | 0.69 ± 0.05 | n.d. |

Values are expressed as means ± standard deviations; n.d. – not detected; α-HCH- α-hexachlorocyclohexane ; β-HCH- β-hexachlorocyclohexane; δ-HCH- δ-hexachlorocyclohexane; M-honey from the Malopolska region; W-honey from the Warmia and Mazury region

**Table S3** The content of PAHs in honey (mg/kg)

| Region of origin | Country of origin | Type | NaP | MeNaP2 | MeNaP1 | Ace | Acp | Flu | Phen | Ant | Fla | Pyr | B[a]a | Chr | B[b]f | B[k]f | B[a]p | I[cd]p | D[ah]a | B[ghi]P |
| --- | --- | --- | --- | --- | --- | --- | --- | --- | --- | --- | --- | --- | --- | --- | --- | --- | --- | --- | --- | --- |
| North Europe | England | Heather I | n.d. | 0.43 ±  0.02 | 0.43 ±  0.01 | n.d. | 7.57 ±  0.48 | n.d. | 6.67 ±  0.27 | 0.86 ±  0.02 | 1.51 ±  0.07 | 1.23 ±  0.04 | 0.14 ±  0.00 | 0.14 ±  0.00 | n.d. | n.d. | n.d. | n.d. | n.d. | n.d. |
|  | England | Heather II | n.d. | n.d. | n.d. | n.d. | 1.45 ±  0.09 | n.d. | 1.45 ±  0.04 | n.d. | 0.72 ±  0.01 | 0.70 ±  0.01 | 0.10 ±  0.00 | 0.08 ±  0.00 | n.d. | n.d. | n.d. | n.d. | n.d. | n.d. |
|  | England | Multiflorous | n.d. | 0.06 ±  0.00 | 0.19 ±  0.01 | n.d. | n.d. | n.d. | 0.99 ±  0.07 | 0.55 ±  0.01 | 0.16 ±  0.00 | 0.14 ±  0.01 | n.d. | n.d. | 0.10 ±  0.00 | n.d. | n.d. | n.d. | n.d. | n.d. |
|  | England | Wildflower | n.d. | 0.15 ±  0.01 | 0.39 ±  0.03 | n.d. | 2.45 ±  0.08 | n.d. | 6.16 ±  0.14 | 0.67 ±  0.02 | 3.56 ±  0.13 | 3.21 ±  0.02 | 0.55 ±  0.03 | 0.77 ±  0.02 | n.d. | n.d. | n.d. | n.d. | n.d. | n.d. |
|  | Scotland | Heather | n.d. | n.d. | 0.16 ±  0.00 | n.d. | 0.78 ±  0.02 | n.d. | 4.19 ±  0.10 | 0.73 ±  0.03 | 1.74 ±  0.08 | 1.64 ±  0.16 | n.d. | n.d. | n.d. | n.d. | n.d. | n.d. | n.d. | n.d. |
|  | Scotland | Multiflorous | n.d. | 0.09 ±  0.00 | n.d. | n.d. | 0.17 ±  0.01 | n.d. | 0.57 ±  0.05 | 0.40 ±  0.01 | 0.18 ±  0.01 | 0.17 ±  0.01 | 0.07 ±  0.01 | 0.07 ±  0.00 | n.d. | n.d. | n.d. | n.d. | n.d. | n.d. |
|  | Scotland | Clover | n.d. | n.d. | n.d. | n.d. | n.d. | n.d. | 0.27 ±  0.00 | 0.26 ±  0.00 | n.d. | 0.10 ±  0.01 | 0.07 ±  0.00 | 0.06 ±  0.00 | n.d. | n.d. | n.d. | n.d. | n.d. | n.d. |
| South Europe | France | Linden | n.d. | 0.51 ±  0.04 | 0.49 ±  0.01 | 0.21 ±  0.01 | n.d. | n.d. | 1.95 ±  0.13 | n.d. | 0.88 ±  0.01 | n.d. | n.d. | 0.35 ±  0.02 | n.d. | n.d. | n.d. | n.d. | n.d. | n.d. |
|  | France | Chestnut | n.d. | 0.16 ±  0.00 | 0.34 ±  0.03 | 0.37 ±  0.02 | 0.09 ±  0.00 | n.d. | 1.32 ±  0.05 | 0.38 ±  0.02 | 1.02 ±  0.03 | 0.82 ±  0.02 | 0.22 ±  0.01 | n.d. | n.d. | n.d. | n.d. | n.d. | n.d. | n.d. |
|  | France | Acacia | n.d. | n.d. | 0.15 ±  0.00 | n.d. | n.d. | n.d. | 0.36 ±  0.02 | 0.27 ±  0.03 | n.d. | n.d. | n.d. | n.d. | n.d. | n.d. | n.d. | n.d. | n.d. | n.d. |
|  | Spain | Lemon blossom | n.d. | 0.14 ±  0.01 | n.d. | 0.06 ±  0.00 | n.d. | n.d. | 0.06 ±  0.01 | 0.51 ±  0.03 | n.d. | n.d. | 0.07 ±  0.01 | 0.04 ±  0.00 | 0.35 ±  0.03 | n.d. | n.d. | n.d. | n.d. | n.d. |
|  | Spain | Heather | n.d. | 0.44 ±  0.03 | 0.35 ±  0.02 | 0.39 ±  0.02 | 0.25 ±  0.02 | n.d. | 3.86 ±  0.06 | 0.46 ±  0.01 | 1.71 ±  0.08 | 1.51 ±  0.07 | 0.54 ±  0.02 | 0.48 ±  0.05 | n.d. | n.d. | n.d. | n.d. | n.d. | n.d. |
|  | Spain | Thyme | n.d. | 0.10 ±  0.01 | n.d. | n.d. | 0.28 ±  0.00 | n.d. | 3.06 ±  0.11 | 0.51 ±  0.02 | 0.24 ±  0.00 | 0.91 ±  0.04 | n.d. | n.d. | 0.11 ±  0.00 | n.d. | n.d. | n.d. | n.d. | n.d. |
|  | Spain | Lavender | n.d. | 0.22 ±  0.01 | 0.30 ±  0.02 | 0.20 ±  0.01 | 0.12 ±  0.00 | n.d. | 3.88 ±  0.26 | 1.01 ±  0.01 | 0.14 ±  0.01 | 1.73 ±  0.01 | n.d. | n.d. | n.d. | n.d. | n.d. | n.d. | n.d. | n.d. |
|  | Spain | Orange blossom | n.d. | n.d. | n.d. | 0.05 ±  0.00 | n.d. | n.d. | 1.26 ±  0.06 | 0.38 ±  0.01 | 1.07 ±  0.11 | 1.10 ±  0.08 | 0.20 ±  0.02 | 0.37 ±  0.03 | n.d. | n.d. | n.d. | n.d. | n.d. | n.d. |
|  | Italy | Eucalyptus | n.d. | n.d. | n.d. | 0.15 ±  0.00 | n.d. | n.d. | 0.60 ±  0.02 | 0.31 ±  0.02 | n.d. | n.d. | n.d. | n.d. | n.d. | n.d. | n.d. | n.d. | n.d. | n.d. |
| East Europe | Slovakia | Multiflorous | n.d. | n.d. | n.d. | n.d. | n.d. | n.d. | 4.81 ±  0.12 | 0.73 ±  0.00 | 1.70 ±  0.06 | 1.29 ±  0.11 | 0.07 ±  0.01 | 0.16 ±  0.01 | n.d. | n.d. | n.d. | n.d. | n.d. | n.d. |
|  | Slovakia | Forest | n.d. | n.d. | 0.18 ±  0.01 | 0.26 ±  0.02 | n.d. | n.d. | 0.52 ±  0.01 | 0.26 ±  0.02 | 0.19 ±  0.00 | 0.14 ±  0.01 | n.d. | n.d. | n.d. | n.d. | n.d. | n.d. | n.d. | n.d. |
|  | Slovakia | Rape | n.d. | 0.07 ±  0.00 | n.d. | 0.11 ±  0.00 | 0.07 ±  0.00 | n.d. | 0.47 ±  0.02 | 0.37 ±  0.02 | 0.15 ±  0.01 | 0.09 ±  0.00 | n.d. | n.d. | n.d. | n.d. | n.d. | n.d. | n.d. | n.d. |
|  | Slovakia | Honeydew | n.d. | n.d. | n.d. | n.d. | n.d. | n.d. | 0.87 ±  0.04 | 0.31 ±  0.01 | 0.35 ±  0.01 | 0.26 ±  0.01 | 0.17 ±  0.01 | n.d. | n.d. | n.d. | n.d. | n.d. | n.d. | n.d. |

| Region of origin | Country of origin | Type | NaP | MeNaP2 | MeNaP1 | Ace | Acp | Flu | Phen | Ant | Fla | Pyr | B[a]a | Chr | B[b]f | B[k]f | B[a]p | I[cd]p | D[ah]a | B[ghi]P |
| --- | --- | --- | --- | --- | --- | --- | --- | --- | --- | --- | --- | --- | --- | --- | --- | --- | --- | --- | --- | --- |
| East Europe | Poland W | Multiflorous | n.d. | 0.06 ±  0.00 | 0.15 ±  0.00 | n.d. | n.d. | n.d. | 0.70 ±  0.03 | 0.39 ±  0.01 | 0.19 ±  0.01 | 0.06 ±  0.01 | n.d. | n.d. | n.d. | n.d. | n.d. | n.d. | n.d. | n.d. |
|  | Poland W | Linden | n.d. | 0.07 ±  0.00 | n.d. | 0.18 ±  0.01 | n.d. | n.d. | 0.45 ±  0.02 | 0.26 ±  0.02 | 0.30 ±  0.01 | 0.17 ±  0.01 | 0.10 ±  0.00 | n.d. | n.d. | n.d. | n.d. | n.d. | n.d. | n.d. |
|  | Poland W | Buckwheat | n.d. | n.d. | n.d. | n.d. | nd | n.d. | 0.49 ±  0.00 | 0.44 ±  0.01 | n.d. | n.d. | n.d. | n.d. | n.d. | n.d. | n.d. | n.d. | n.d. | n.d. |
|  | Poland M | Multiflorous | n.d. | 0.07 ±  0.00 | 0.07 ±  0.00 | n.d. | 0.16 ±  0.01 | n.d. | 1.94 ±  0.12 | 0.23 ±  0.02 | 1.38 ±  0.07 | 1.28 ±  0.07 | 0.27 ±  0.05 | 0.28 ±  0.02 | n.d. | n.d. | n.d. | n.d. | n.d. | n.d. |
|  | Poland M | Linden | n.d. | n.d. | n.d. | 0.24 ±  0.00 | n.d. | n.d. | 0.50 ±  0.04 | 0.24 ±  0.02 | 0.18 ±  0.01 | 0.16 ±  0.01 | n.d. | n.d. | n.d. | n.d. | n.d. | n.d. | n.d. | n.d. |
|  | Poland M | Rape | n.d. | 0.11 ±  0.00 | n.d. | 0.04 ±  0.00 | n.d. | n.d. | 4.03 ±  0.24 | 0.71 ±  0.02 | 1.46 ±  0.01 | 1.11 ±  0.07 | 0.18 ±  0.01 | n.d. | 0.41 ±  0.03 | n.d. | n.d. | n.d. | n.d. | n.d. |

Values are expressed as means ± standard deviations; n.d. – not detected; NaP – naphthalene; MeNaP2 – 2-methylnaphthalene; MeNaP1 – 1-methylnaphthalene; Ace – acenaphthene; Acp – acenaphthylene; Flu – fluorene; Phen - phenanthrene; Ant - anthracene; Fla – fluoranthene; Pyr – pyrene; B[a]a – benzo[a]anthracene; Chr – chrysene; B[b]f – benzo[b]fluoranthene; B[k]f – benzo[k]fluoranthene; B[a]p benzo[a]pyrene; I[cd]p – indeno[1,2,3-c,d]pyrene; D[ah]a – dibenzo[a,h]anthracene; B[ghi]P – benzo[g,h,i]perylene; Poland M-honey from Malopolska region; W-honey from Warmia and Mazury region
